# Universality of slip avalanches in flowing granular matter

**DOI:** 10.1038/ncomms10641

**Published:** 2016-02-17

**Authors:** D. V. Denisov, K. A. Lörincz, J. T. Uhl, K. A. Dahmen, P. Schall

**Affiliations:** 1 Institute of Physics, University of Amsterdam, PO Box 94485, 1090 GL Amsterdam, The Netherlands; 2 Department of Physics, University of Illinois at Urbana Champaign, 1110 West Green Street, Urbana, Illinois 61801, USA

## Abstract

The search for scale-bridging relations in the deformation of amorphous materials presents a current challenge with tremendous applications in material science, engineering and geology. While generic features in the flow and microscopic dynamics support the idea of a universal scaling theory of deformation, direct microscopic evidence remains poor. Here, we provide the first measurement of internal scaling relations in the deformation of granular matter. By combining macroscopic force fluctuation measurements with internal strain imaging, we demonstrate the existence of robust scaling relations from particle-scale to macroscopic flow. We identify consistent power-law relations truncated by systematic pressure-dependent cutoff, in agreement with recent mean-field theory of slip avalanches in elasto-plastic materials, revealing the existence of a mechanical critical point. These results experimentally establish scale-bridging relations in the flow of matter, paving the way to a new universal theory of deformation.

## Abstract (web summary)

Whether the deformation of amorphous materials is governed by universal scaling, a characteristic feature of the critical phenomena, is currently under debate. Here, Denisov *et al*. provide experimental evidence by linking the internal strains at microscales to the fluctuations in the applied force.

## Graphical abstract

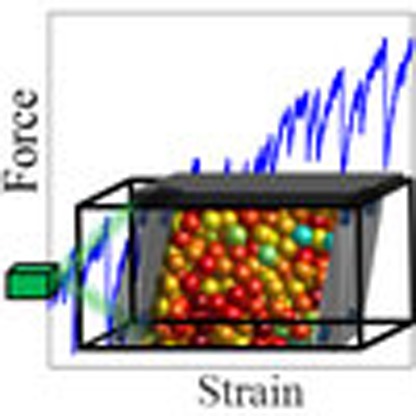

Unifying scaling relations in the deformation of solids have been a long-standing challenge of material science and engineering. Such universal scaling relations are very attractive, as they provide a naturally scale-bridging framework connecting macroscopic stress–strain response to microscopic single-particle fluctuations in a single theory of deformation. Recent work on amorphous materials highlights generic features of the flow, mechanics and microscopic dynamics of a broad range of materials from metallic glasses to soft glasses^1^,^2^,^3^,^4^,^5^ to granular^6^,^7^,^8^ and crystalline^9^,^10^,^11^ matter, lending credence to the idea of a universal theory of deformation^12^. While scaling approaches^13^ that describe the scale-free flow of materials have been developed recently, direct experimental proof of an underlying internal scaling of strain over the full-length-scale range remains elusive. This would require linking the macroscopic applied force and its fluctuations to the microscopic internal fluctuations of plasticity on length scales from system size down to particle scale, a highly challenging task prohibitively difficult in conventional atomic solids.

Granular and soft materials offer the advantage that the internal flow field can be imaged conveniently with optical techniques, providing access to important microscopic quantities such as internal displacements and strain fields, which are hardly accessible in molecular systems. In particular, granular matter with particle sizes of the order of millimetres allows the motion of individual particles to be tracked accurately using bulk imaging techniques^14^,^15^,^16^. At the same time, macroscopic stress–strain measurements allow the applied force and its fluctuations to be monitored with exquisite time resolution. The combination of both offers a unique experimental opportunity to bridge length scales from single particle to macroscopic scale, making granular materials prime candidates to elucidate generic, scale-bridging aspects of flow.

A recent mean-field model^13^ provides a universal description of material flow in terms of slip avalanches coupled by their induced internal elastic field: the material slips locally at weak spots, which are elastically coupled to other weak spots throughout the material resulting in highly correlated slip avalanches. The resulting scale-bridging model offers a promising generic framework to bridge particle-scale dynamics to macroscopic stress–strain response. Recent highly sensitive force measurements on crystalline nanopillars^17^ and amorphous metals^7^ have indeed detected signatures of these scaling relations in the applied force fluctuations; however, the crucial internal scaling relations bridging down to microscopic displacements remain experimentally elusive, and the validity of this generic concept remains unclear. Furthermore, the universality of this approach, that is, its generality and applicability to a wide range of materials including soft and granular matter remains an open issue.

Here, we provide the first direct experimental measurement of just these scale-bridging relations in the flow of granular matter. By combining macroscopic force measurements with direct imaging of the internal strain distribution, we unveil surprisingly consistent scaling relations from macroscopic force response down to microscopic fluctuations. We demonstrate that at all scales, fluctuations exhibit consistent power-law distributions and correlations, truncated by systematic cutoff that grows with the applied confining pressure. These measurements give strong experimental evidence of the proximity of a mechanical critical point. We show that these scaling relations agree with predictions of the universal mean-field theory of slip avalanches in elasto-plastic materials^13^. These results for the first time demonstrate experimentally the existence of robust internal scaling relations in granular flow, bridging from microscopic strain to macroscopic stress and accounted for by a universal mean-field theory. Hence, these measurements lay the ground for a generic description of material flow within a universal, scale-bridging framework.

## Results

### Force fluctuations

**Figure 1:**
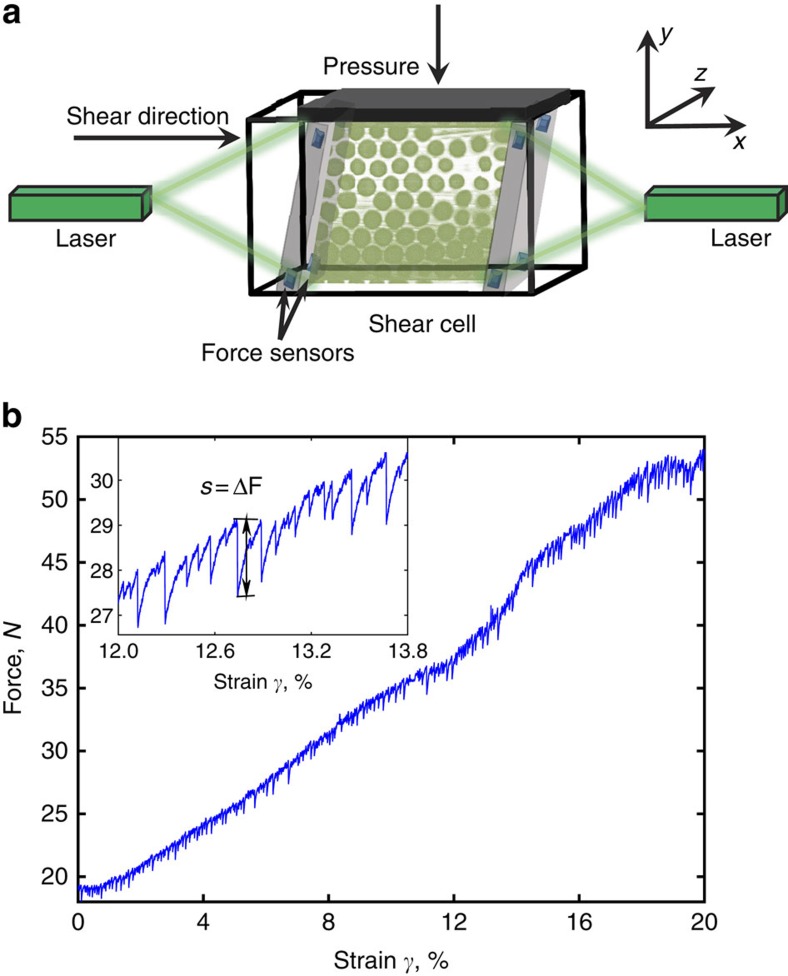
Measurement of force fluctuations and internal imaging. (**a**) Schematic of the shear cell set-up with laser sheet imaging. Two lasers with cylindrical lenses aligned opposite of each other illuminate thin sections of the sheared suspension for imaging of the individual particles. Force sensors in the corners of the shearing walls record the applied shear force. Loads added on top exert a constant confining pressure. (**b**) Measured force as a function of applied strain shows pronounced fluctuations. The maximum detected fluctuation is ∼3 N, while the noise level of the fluctuations is ∼10^−2^ N, leading to a total range of observable fluctuations of around 2.5 orders of magnitude. Inset: 10-time magnification of these force data shows several small and large stress releases.

To combine both macroscopic and microscopic measurements, we use a shear-cell set-up that links force measurements by built-in pressure sensors with simultaneous particle-scale internal imaging by laser sheets (Fig. 1a). This allows us to track fluctuations of the applied force, while imaging the internal strain distribution over the full-granulate volume (Methods section). We do this as a function of applied load that exerts a well-defined confining pressure on the top layer of the granulate. By tilting the side walls, we shear the granulate uniformly at constant (low) rate up to a maximum strain of 0.2, starting from a well-defined initial state. To increase statistics, we average over 10 shear cycles.

**Figure 2:**
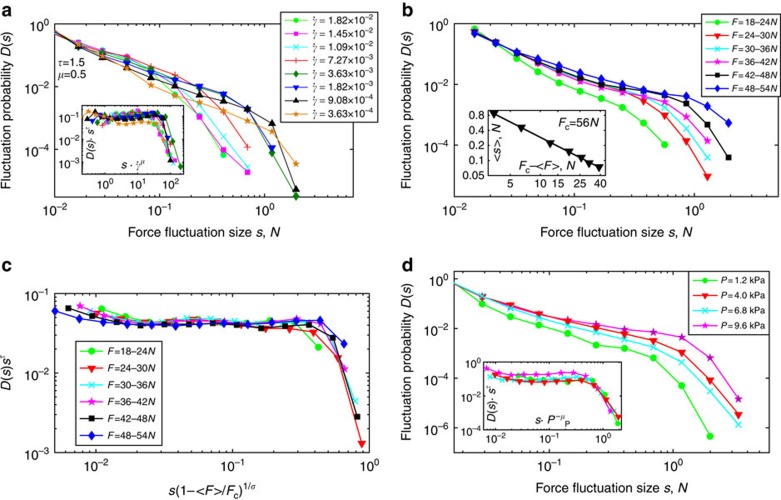
Scaling of force fluctuations. (**a**) Probability distribution, *D*(*s*), as a function of force fluctuation size, *s*, for different shear rates (see legend) at constant confining pressure *P*=9.6 kPa. These data suggests truncated power laws 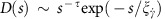 with 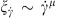. Inset shows these data collapse for *τ*=1.5 and *μ*=0.5. (**b**) Force dependence of the size distribution (see legend) at constant confining pressure *P*=9.6 kPa and shear rate 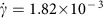 s^−1^. Inset: mean avalanche size as a function of force difference *F*_c_−*F*. The double-logarithmic plot indicates divergence 〈*s*〉∝(*F*_c_−*F*)^*α*^ with *α*=−1.02 on approaching *F*_c_=56 *N*. (**c**) Data collapse of the force-dependent avalanche size distribution shown in **b**, 
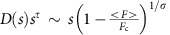
, with *τ*=1.5, *F*_c_=56 and *σ*∼0.6. (**d**) Size distribution *D*(*s*) for different confining pressures (see legend) at constant shear rate 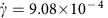 s^−1^. Inset shows data collapse 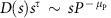, with *τ*=1.5 and *μ*_P_=0.4.

Using this set-up, we can detect pronounced force fluctuations as shown in Fig. 1b: sharp force drops follow continuous force increases, revealing internal relaxations of the granulate that release some of the applied force. We define a single relaxation event from a monotonic force drop with amplitude >10^−2^ N and short duration <10^−1^ s. Using this definition, we detect ∼10^4^ relaxation events in a total sequence of 10 shear experiments. We define their size *s* from the magnitude Δ*F* of a sharp force drop (see Fig. 1b inset). Plotting the relative frequency, *D*(*s*), as a function of size *s* reveals power-law distributions *D*(*s*)∼*s*^−*τ*^ truncated by systematic strain-rate dependent cutoff as shown in Fig. 2a. This cutoff moves to smaller size as the shear rate increases, suggesting that fluctuations may overlap and a relaxation event cannot complete before a new one starts, leading to truncation of large relaxations according to 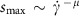 (ref. 18). Indeed, we can collapse all data by rescaling avalanche sizes by 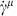, with *μ*=0.5, as shown in Fig. 2a, inset. Furthermore, all curves exhibit a robust scaling exponent of *τ*∼1.5, as is demonstrated by the collapse in the inset. The scaling collapse applied here and below allows extrapolation of critical behaviour away from the critical point; this is particularly useful in finite-size systems, where correlation lengths close to the critical point would exceed the system size^17^,^19^.

Our measurements probe the initial loading of the granulate, where the applied force increases steadily with strain. This allows us to investigate how the fluctuations depend on the applied force magnitude. We sort fluctuations by the increasing force magnitude and plot separate distributions in Fig. 2b. Clearly, all distributions exhibit consistent power-law decay with a cutoff that grows with the applied force magnitude. To evaluate this growth of fluctuations, we compute average avalanche sizes <*s*> that are less affected by the poor statistics at large *s*. These grow monotonically with the applied force magnitude as shown in Fig. 2b, inset. We find a power-law dependence with exponent −1 on approaching the critical force *F*_c_∼56±3 N, as shown by the double-logarithmic representation of <*s*> as a function of *F*−*F*_c_. This critical force is consistent with the apparent saturation value of the force at large strain as shown in Fig. 1b. Using this value of *F*_c_ we can indeed collapse the force-dependent data shown in Fig. 2b by rescaling the avalanche sizes by (1−<*F*>/*F*_c_)^1/*σ*^, with *σ*≈0.6 as shown in Fig. 2c. This scaling collapse, which extrapolates the critical behaviour close to the critical point, indicates that the granular flow develops truly critical behaviour on approaching *F*_c_.

To explore this critical behaviour in more detail, we vary the rigidity of the granulate by changing the applied load. By releasing some of the confining pressure, we lower the rigidity of the granulate^20^,^21^,^22^,^23^, and we expect smaller stress build-up and hence smaller force fluctuations to occur^22^,^24^. This is indeed what we find as shown in Fig. 2d: as the confining pressure decreases, the cutoff of the power-law shifts to smaller sizes, indicating smaller fluctuations, similar to the applied shear force dependence in Fig. 2b. We can collapse all curves using the rescaling 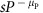 with *μ*_P_=0.4 (Fig. 2d, inset). A similar exponent is observed when we collapse the avalanche sizes (Fig. 2b) with respect to the growing applied force, where we obtain *μ*_F_∼0.5 suggesting that the dependence on both shear force magnitude and confining pressure can be accounted for by similar scaling relation. This consistent critical scaling suggests a simple scaling model to account for the observed force fluctuations.

We apply recent mean-field theory of a stick-slip model of deformation^8^, where critical behaviour arises from the interplay of local slip and long-range elastic interactions. The model assumes that the material has weak spots where it slips when the stress exceeds a local failure stress. Because all weak spots are elastically coupled, a slipping weak spot can trigger other weak spots to also fail, resulting in a slip avalanche^13^. In the quasistatic limit, where the material is sheared slowly enough so that every avalanche has time to complete, the model predicts that the probability density distribution *D*(*s*, *F*) of slip sizes *s* occurring at an applied force *F* follows a power-law with a force-dependent maximum size cutoff *s*_max_∝(*F*_c_−*F*)^−1/*σ*^, hence,


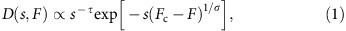


where, *F*_c_ is a critical force, above which the material cannot sustain any load. The exponents *τ*=3/2 and *σ*=1/2 are detail-independent ‘universal' scaling exponents. At finite shear rate, avalanche overlap leads to truncation of large avalanches according to 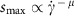 with *μ*=2 for steady-state flow. The predicted exponents *τ*=3/2 and *σ*=0.5 are indeed in good agreement with our observations (Fig. 2), while the value *μ*=2 predicted for steady-state deviates substantially from the measured value *μ*=0.5; such deviations are, however, expected when the experiments are not in steady state, as is the case here and in ref. 7. Yet, the increasing applied force magnitude allows us to test the predicted force dependence of slip sizes. To do so, we use *D*(*s*, *F*) from equation (1) to compute average slip sizes <*s*>=∫*s D*(*s*, *F*)d*s*, obtaining <*s*(*F*)>∝(*F*_c_−*F*)^(*τ*−2)/*σ*^ (ref. 7). The model thus predicts a power-law dependence with exponent (*τ*−2)/(*σ*)=−1. The slope of the data in Fig. 2b (inset) is *α*∼−1.02, in excellent agreement with this prediction. The critical force *F*_c_=56±3*N* is also very reasonable, given that the applied force in our measurements *F*<55*N*. Thus, overall the agreement with this simple model is remarkable.

### Internal strain imaging

**Figure 3:**
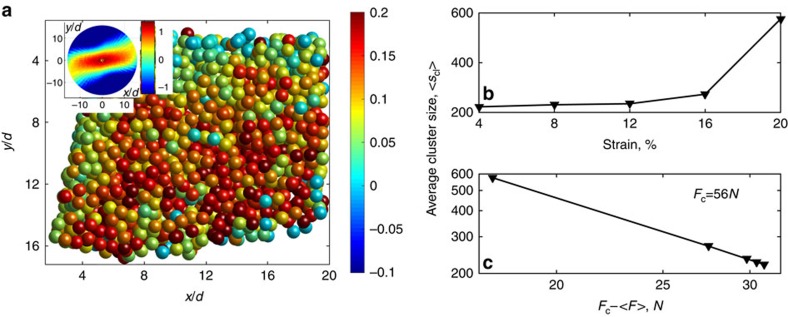
Microscopic strain distribution. (**a**) Reconstruction of 8-mm-thick section of the sheared granulate shows the distribution of shear strain 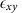 accumulated over 20% strain for the same pressure and shear rate as in Fig. 2b. Inset: Angle-resolved correlation function of the fluctuations of 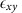; correlation value is indicated with colour, see colour bar. (**b**) Growth of average cluster size 〈*s*_cl_〉 with strain. Cluster consists of highly active particles. (**c**) The double-logarithmic plot indicates divergence 〈*s*_cl_〉∝(*F*_c_−*F*)^*β*^ at *F*_c_=56 N (*β*≈−1.46).

The advantage of using granular matter to elucidate scale-bridging relations is that we can directly image the internal strain distribution. Using laser sheets, we visualize individual particles in the bulk of the granulate and track their motion over the entire strain cycle. We then determine, for each particle, the local strain from the displacement of the particle relative to its nearest neighbours (Methods section). A two-dimensional rendering of the most relevant strain component 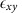 is shown in Fig. 3a, where colour indicates the magnitude and sign of 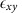, see colour bar. High shear strain concentrates in connected clusters that span the imaged volume, indicating correlated slip events accumulated over time. However, the deformation remains overall homogeneous and no shear banding occurs, as dictated by the rigid tilting walls, see Fig. 1a. We focus on the top 20% highest strain particles and follow them as a function of strain using fixed strain intervals of 4% to capture always the incremental strain. Plotting the average cluster size <*s*_cl_> of high-strain regions as a function of applied strain (Fig. 3b) clearly reveals that the clusters grow with the applied strain. When we plot <*s*_cl_> as a function of the applied force, we find indeed a power-law divergence on approaching the critical force *F*_c_∼56 N, similar to the average avalanche size in Fig. 2b inset, as shown by the double-logarithmic representation of <*s*_cl_> versus *F*−*F*_c_ in Fig. 3c. This behaviour is robust within reasonable limits of the threshold strain used to define the high-strain particles. This direct correspondence to the avalanche size with the same value of the critical force gives independent microscopic evidence of the increasingly critical state on approaching *F*_c_. We further investigate the structure of these highly active clusters. We compute, for each cluster, the radius of gyration 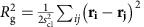 from relative particle positions **r**_**i**_−**r**_**j**_ in the cluster, and determine the scaling of *R*_g_ as a function of cluster size *s*_cl_. We find that the gyration radius scales with cluster size as 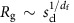, with the fractal dimension *d*_f_=2.5±0.1, which again remains robust on varying the threshold strain and considering up to the top 2% high-strain particles. This value lies very close to the value 2.53 of three-dimensional (3D) percolation, suggesting that the accumulated avalanches span the entire system.

**Figure 4:**
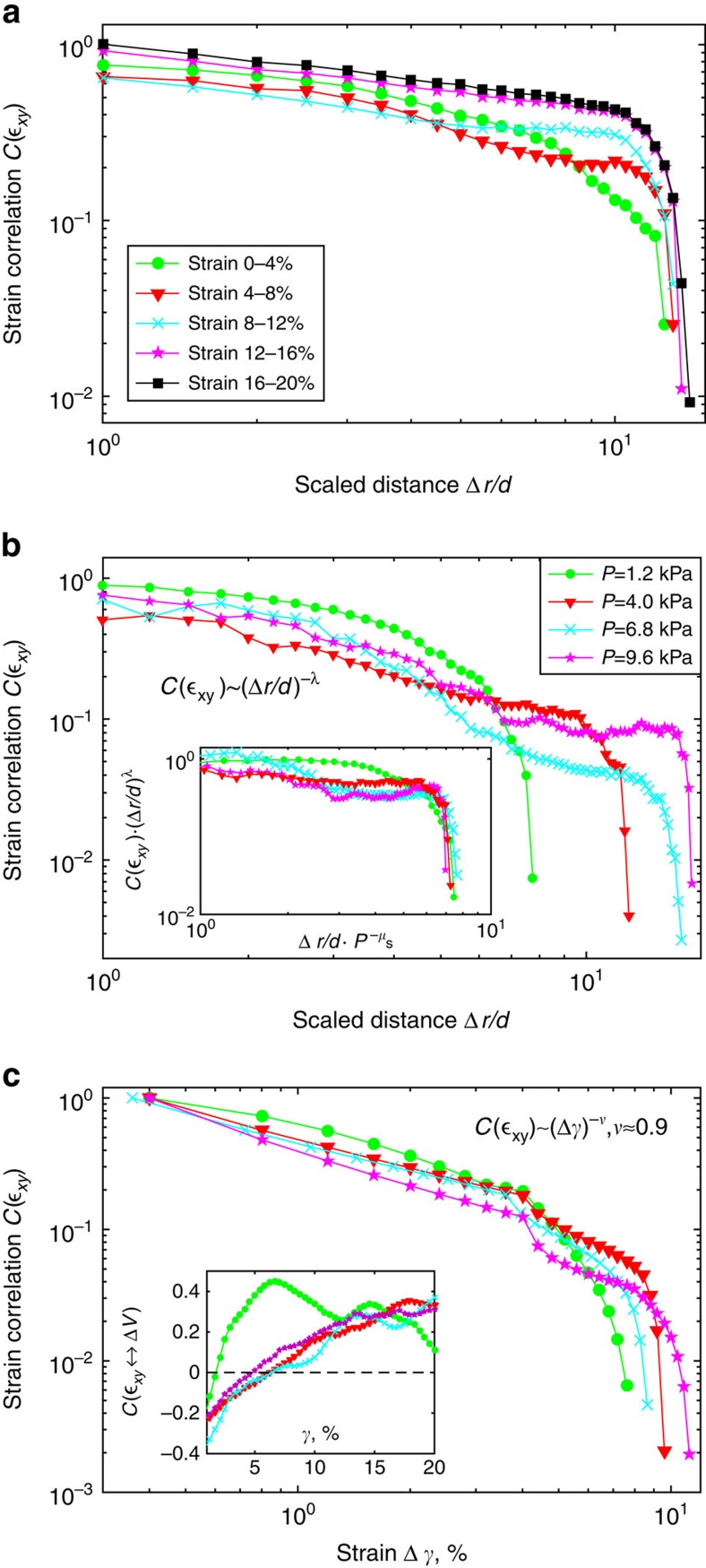
Strain correlation and scaling. (**a**) Decay of strain correlations along the shear direction with increasing strain at fixed intervals 4% as in Fig. 3b. Correlation length grows with increasing strain. (**b**) Decay of strain correlations. These data suggests 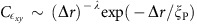 with 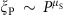. Inset shows data collapse obtained for *λ*=0.8 and *μ*_S_=0.4. (**c**) Autocorrelation of particle strain activity as a function of applied strain. Colour and symbols correspond to the confining pressures in **b**. These data suggests 
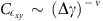
 with *ν*≈0.9. Inset shows correlation between the shear strain component 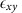 and dilatation, averaged over all particles.

To get full insight into the microscopic avalanche evolution, we measure directly the internal scaling relations of strain using the spatial correlation function 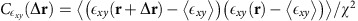 that correlates strains at locations separated by **Δr**. Here, angular brackets denote averaging over all particles and 
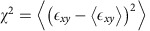
, the s.d. The resulting correlation functions are anisotropic, exhibiting the longest correlation length along the shear direction, as shown in the inset of Fig. 3a. This correlation function visualizes directly the correlations between slip events that underlie the collective avalanches. Along the shear direction, the correlations decay closely to a power-law with cutoff that grows with the applied strain, see Fig. 4a. This behaviour is analogous to that of the force fluctuations, where the avalanche size grows with force magnitude (Fig. 2b).

The increase of the correlation length is even more pronounced with growing confined pressure—at the largest pressure, the correlation length grows up to the full-system size, see Fig. 4b. Such increase of power-law cutoff is in qualitative agreement with the growth of force fluctuations in Fig. 2d. To test this quantitatively, we determine the full-correlation volume. We observe that in the other two spatial directions, correlation lengths hardly change; consequently, the growth shown in Fig. 4b reflects directly the bulk growth of the avalanches. Hence, if it is indeed the growth of these internal correlations that underlies the growth of macroscopic force fluctuations, we expect the same scaling collapse to apply to both. This is precisely what we find when we rescale Δ*r*/*d* in the shear direction by 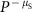 (inset of Fig. 4b): excellent collapse is obtained for *μ*_S_=0.4, the same exponent as for the macroscopic force fluctuations, demonstrating their common origin. We hence identify experimentally the underlying mechanism behind the scaling of force fluctuations: in the highly constrained granular material, microscopic strain fluctuations are strongly correlated, leading to power-law correlations from particle scale to system size for the largest confining pressure. The slope of this power-law correlation, *λ*∼0.8, is indeed reasonably close to the mean field value *λ*=1 along the direction of greatest slip^25^. It is also close to the numerical value obtained in simulations on Lenard-Jones systems in quasistatic shear^5^ that report *λ*=1.18 along the direction of greatest displacement. These results demonstrate the robust internal scaling of microscopic strain underlying the macroscopic fluctuations, consistent with the mean-field model.

Surprisingly, we observe also strong time correlation of the microscopic deformation, in addition to its spatial correlation. To investigate this in detail, we determine the typical persistence time of the strain activity of a particle. We correlate, for each particle, its instantaneous strain with that at a later time to compute 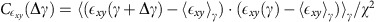 with 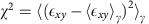. The resulting time correlation function (Fig. 4c) approaches a power-law decay, again truncated by a pressure-dependent cutoff. Remarkably, the activity of a particle is correlated over half of the straining cycle, indicating strong memory. Such memory can arise from local shear-induced dilation^26^ that facilitates successive shear events. We investigate this shear-dilation coupling by using the full-strain tensor to compute the local dilation from the normal strains according to 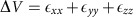. The resulting normalized correlation coefficient between shear and dilation shows indeed significant coupling (Fig. 4c, inset), indicating that shear-dilation coupling plays an important role in the observed internal time coherence.

## Discussion

In conclusion, our scale-bridging measurements provide the first direct experimental evidence of critical internal scaling relations behind the critical scaling of force fluctuations in flowing particulate matter. These scaling relations have the form of those known from the physics of critical phenomena: the correlation length grows with increasing applied force to diverge at the critical force *F*_c_, where the material yields. Hence, macroscopic critical fluctuations originate from a hierarchical internal strain distribution with diverging correlation length. While the granular system allows us to conveniently image and measure these scaling relations, they should be generic to the deformation of solid matter including both amorphous^7^ and crystalline materials^27^, as the model is generic, based only on elasticity and local failure. The excellent agreement of the model predictions with our scale-bridging measurements lays the ground for a new understanding of yielding and flow central to fields from engineering to material science to geology, delineating a new universal theory of plastic deformation.

## Methods

### Experiments

As granular system, we use polymethyl methacrylate spheres with diameter of *d*=1.5 mm and polydispersity of ∼5%. We fill the particles into a shear device with transparent, tiltable side walls with built-in pressure sensors (Fig. 1a). The shear cell has dimensions of 10 × 10 × 10 cm^3^, containing about 3 × 10^5^ particles. A top plate charged with additional weights is used to confine the granulate vertically, exerting a constant normal force between 10 and 100 N on the top layer of the granulate. After a fixed pre-shear protocol generating a reproducible initial packing, the granulate is sheared at a constant rate between 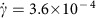 and 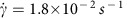 to a total strain of *γ*=20%. We measure the applied force at a frequency of 500 Hz with an accuracy of ±10^−2^ N, and a maximum force on each sensor of 55 N. To visualize the motion of the individual granular particles we use particles with a diameter of 4 mm, and add an index-matching solution of water, NaI and fluorescein that makes the particles appear as dark spots on a bright background^14^. Individual particles are imaged in a 100 mm by 70 mm by 85 mm volume using laser sheets (Fig. 1a). 3D image stacks consisting of 180 sections with a separation of 0.389 mm are acquired every 70 s during the entire shear cycle.

### Analysis

To reconstruct particle positions and trajectories from 3D image stacks we use algorithms based on^28^ to track particle centres with an accuracy of ±0.01 mm in the *x*- and *y*-, and ±0.03 mm in the *z* direction^14^. To determine the local strain, we follow individual particle trajectories and identify the nearest neighbours of each particle as those that are in a range corresponding to the first minimum of the pair correlation function. This range corresponds to 1.5 particle diameters. We then compare the nearest neighbor vectors **d**_**n**_=**r**_**n**_−**r**_**0**_, at applied strains of *γ* and *γ*+Δ*γ*. Here, the vector **r_0_** indicates the position of the central particle and the index *n* refers to the nearest-neighbour particles. We then find the best affine deformation tensor 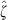 that transforms the nearest-neighbour vectors over the strain interval Δ*γ* by minimizing the mean square difference 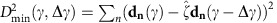 (ref. 29), where 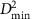 represents the local non-affine deformation. The symmetric part of 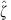 corresponds to the strain tensor 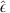 of the central particle. We concentrate our analysis on the shear direction—shear gradient (*x*−*y*) plane, taking into account the tensor component 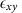, on which we base our correlation function analysis, see Fig. 4c. To correlate shear and dilation components, we take 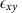 and the sum of the three normal strains, 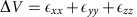 as measure of local dilation. We then compute correlations according to 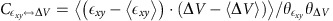, where 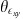 and *θ*_Δ*V*_ denote s.d.'s of 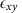 and Δ*V*, and angular brackets denote averaging over all particles.

To determine force fluctuations from the measured force data, we identify changes with respect to the monotonically increasing force, see Fig. 1. Stress relaxation events correspond to sharp force drops; we thus take into account only monotonic events with negative force derivative and short duration <10^−1^ s. We take the magnitude of these abrupt force changes to be the size *s* of a relaxation event. The size probability distribution then follows a power-law *D*(*s*)∼*s*^*−τ*^, as shown in Fig. 2a. To demonstrate the universality of the distributions, we collapse them onto master curves by scaling the abscissa with 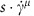 and the ordinate axis with *D*(*s*)·*s*^*τ*^, see insets of Fig. 2a.

## Additional information

**How to cite this article:** Denisov, D. V. *et al*. Universality of slip avalanches in flowing granular matter. *Nat. Commun.* 7:10641 doi: 10.1038/ncomms10641 (2016).
